# Idiopathic (primary) achalasia

**DOI:** 10.1186/1750-1172-2-38

**Published:** 2007-09-26

**Authors:** Farnoosh Farrokhi, Michael F Vaezi

**Affiliations:** 1 Division of Gastroenterology and Hepatology, Center for Swallowing and Esophageal Disorders, Vanderbilt University Medical Center, Nashville, Tennessee, USA

## Abstract

Idiopathic achalasia is a primary esophageal motor disorder characterized by esophageal aperistalsis and abnormal lower esophageal sphincter (LES) relaxation in response to deglutition. It is a rare disease with an annual incidence of approximately 1/100,000 and a prevalence rate of 1/10,000. The disease can occur at any age, with a similar rate in men and women, but is usually diagnosed between 25 and 60 years. It is characterized predominantly by dysphagia to solids and liquids, bland regurgitation, and chest pain. Weight loss (usually between 5 to 10 kg) is present in most but not in all patients. Heartburn occurs in 27%–42% of achalasia patients. Etiology is unknown. Some familial cases have been reported, but the rarity of familial occurrence does not support the hypothesis that genetic inheritance is a significant etiologic factor. Association of achalasia with viral infections and auto-antibodies against myenteric plexus has been reported, but the causal relationship remains unclear. The diagnosis is based on history of the disease, radiography (barium esophagogram), and esophageal motility testing (esophageal manometry). Endoscopic examination is important to rule out malignancy as the cause of achalasia. Treatment is strictly palliative. Current medical and surgical therapeutic options (pneumatic dilation, surgical myotomy, and pharmacologic agents) aimed at reducing the LES pressure and facilitating esophageal emptying by gravity and hydrostatic pressure of retained food and liquids. Although it cannot be permanently cured, excellent palliation is available in over 90% of patients.

## Background

### Definition and epidemiology

Idiopathic (primary) achalasia is a primary esophageal motor disorder of unknown etiology characterized by esophageal aperistalsis and abnormal lower esophageal sphincter (LES) relaxation in response to deglutition [1-4]. It is a rare disease with an annual incidence of approximately 1/100,000 and a prevalence rate of 10/100,000 [5], but it is frequent enough to be encountered at least once by every gastroenterologist.

### History

Achalasia was first described and termed by Sir Thomas Willis in 1674, where he suggested that the disease is due to the loss of normal inhibition in the distal esophagus [6]. Since then, the development of new diagnostic techniques stimulated new ideas on the etiology and pathophysiology of the disease leading to various theories in identifying the nature of motor disturbances in esophageal regions. However, the initiating cause is still elusive [3,6,7].

Studies have shown a similar rate for achalasia in men and women. The disease is characterized predominantly by dysphagia to solids and liquids, bland regurgitation, and chest pain. The diagnosis of achalasia is relatively easy to make with a good history, radiography (barium esophagogram), and esophageal motility testing (esophageal manometry). Endoscopic examination is important to rule out malignancy as the cause of achalasia [8].

Since neuronal degeneration is irreversible, treatment of achalasia is strictly palliative. Current medical and surgical therapeutic options proposed for this well-recognized motor disorder of esophagus are aimed at reducing the LES pressure and facilitating esophageal emptying by gravity and hydrostatic pressure of retained food and liquids [8-12]. These therapeutic options include pneumatic dilation, surgical myotomy, and less effectively pharmacologic agents [11,13].

In this review article we detail on the pathogenesis, etiology, diagnosis, and treatment options for this motor disorder of esophagus.

## Clinical features

Achalasia is one of the most investigated motor disorders of the esophagus [4,7]. The disease can occur at any age but it is usually diagnosed between 25 and 60 years. Progressive dysphagia to solids followed by liquids (82%–100%) is the first clinical symptom of achalasia [13]. Although dysphagia can occur in patients with other esophageal motility disorders, this symptom is most characteristic of achalasia and strongly suggests the diagnosis.

Regurgitation not responding to proton pump inhibitor (PPI) therapy and weight loss can be seen in between 30% to 90% of the patients. Regurgitation of material retained in the dilated esophagus, especially during supine position at night, may lead to aspiration. Weight loss (usually between 5 to 10 kg) is not present in all patients.

Chest pain is another presenting symptom of achalasia (17%–95%). The occurrence of this symptom is unrelated to the LES pressure [4,13]. Chest pain seems to be the only symptom that is affected by the age and gender [4,14-16], as chest pain has been reported to be 1.7 more frequent in women than in men with achalasia [15].

Heartburn, the main symptom of gastroesophageal reflux disease (GERD), may also occur infrequently (27%–42%) in achalasia patients. Although the mean LES pressure in patients with achalasia who experience heartburn has been reported to be significantly lower than that in patients without the symptom [17], this symptom is most likely related to the production of lactic acid from retained foods or exogenous ingested acidic materials such as carbonated drinks [1].

Difficulty belching has also been reported in up to 85% of the patients, and is due to a defect in relaxation of the upper esophageal sphincter in these patients.

The information on cancer risk in achalasia is insufficient. There are many studies on this and the great majority of them suggests a significantly increased risk [18]; however, there are currently no recommendations for surveillance of achalasia patients for esophageal cancer.

## Pathophysiology of idiopathic achalasia

Distal esophageal wall and LES are innervated by postganglionic neurons, consisting of excitatory and inhibitory neurons. The excitatory neurons release acetylcholine while the inhibitory neurons release nitric oxide (NO) and vasoactive intestinal polypeptide (VIP), resulting in esophageal and LES contractions and relaxations, respectively [3].

The NO and VIP releasing inhibitory neurons are the target in idiopathic achalasia. Loss of these inhibitory neurons due to either intrinsic or extrinsic causes will result in the manometric consequence of failure of LES relaxation as well as loss of esophageal peristalsis [3,4,19].

Several studies on humans and animals [20,21] have suggested that extrinsic causes such as lesions located in the central nervous system (CNS) may produce manometric findings of achalasia. Abnormalities of the vagal nerve fibers outside the CNS has also been associated with achalasia; however, extrinsic innervation abnormalities are rare findings in achalasia patients [22-24] and are thus probably not the primary mechanism of the disease.

In contrast, intrinsic loss of inhibitory myenteric neurons in both the esophagus and LES in patients with achalasia has been reported as the most likely contributory factor in the pathophysiology of achalasia. Studies [25-27] have suggested that loss of VIP and NO secreting neurons leads to an imbalance between the excitatory and inhibitory neurons of the myenteric plexus, producing irreversible manometric changes in such patients. Morphologic studies of the esophageal myenteric plexus have also confirmed the loss of myenteric ganglion cells in achalasia [28,29]. In such studies, loss of ganglion cells was associated with inflammation and, even in severe cases, the myenteric nerves had been replaced by collagen.

## Etiology of idiopathic achalasia

### Familial

The existence of familial cases suggest that achalasia is an inherited disease [30-33]. Such familial cases have been mostly seen in the pediatric population, between siblings and in a few cases in monozygotic twins [30,31]. There are also a few reports of a parent-child association for achalasia [32]. Although these evidences suggest an autosomal recessive mode of inheritance for this disease [30,33], the rarity of familial occurrence does not support the hypothesis that genetic inheritance is a significant etiologic factor. Instead, it is proposed that genetic predisposition in such individuals probably increases their susceptibility to acquiring achalasia after exposure to common environmental factors that may play a role in the pathogenesis [3].

### Infection

Several studies have suggested a possible association between viral infections and achalasia [34,35]. In such studies, various viral antibodies were measured in sera of the patients with achalasia and the normal controls, and only measles and varicella zoster virus antibodies were found to be higher among a number of achalasia patients. On the other hand, in the clinical setting not all patients with measles and varicella will develop achalasia [3]. Using polymerase chain reaction, other studies [36,37] have demonstrated no evidence of any viral products in the esophageal tissue of patients with achalasia. In addition, even those studies that found evidence of a virus, could not establish a causal relationship [3], thus, the infectious etiology of achalasia remains an unclear matter.

### Autoimmune

Increased prevalence of circulating antibodies against myenteric plexus in some achalasia patients led to the suggestion of a role for auto-antibodies in the pathogenesis of this disease [38,39]; however, a recent study by Moses *et al *[40] suggested that these circulatory antibodies are most likely the result of a nonspecific reaction to the disease process instead of being the cause of the disease. This idea was supported by detection of similar antibodies in patients without achalasia.

Ultra-structural studies [41,42] of the esophageal tissue of patients with achalasia have also found inflammatory infiltrates around myenteric neurons, while in control group normal myenteric plexus was found without infiltration.

Multiple case-control studies [43-46] have reported a significant association with HLA class II antigens in idiopathic achalasia. The most recent study [46] also showed that achalasia patients with associated HLA allele were found to have higher prevalence of circulating antimyenteric autoantibodies, which supported the autoimmune etiology [3]. HLA association also suggests immunogenetic predisposition for idiopathic achalasia; however, this should be taken with caution as not all the achalasia patients have associated HLA antigens.

## Diagnosis and differential diagnosis

The diagnosis of idiopathic achalasia is relatively straight forward with a well-documented medical history, radiography, and esophageal motility testing.

### History

In the early stages of the disease, dysphagia may be very subtle and can be misinterpreted as dyspepsia, poor gastric emptying, or stress. The presence of heartburn due to food stasis can add to this confusion. As the disease progresses, difficulty swallowing characteristically occurs with both solid foods, and liquids. The dysphagia is more to solids than liquids. To ease progression of the food bolus, patients usually modify their eating habits: eating more slowly or use certain maneuvers such as raising the arms, or arching the back.

### Esophageal manometry

**Figure 1:**
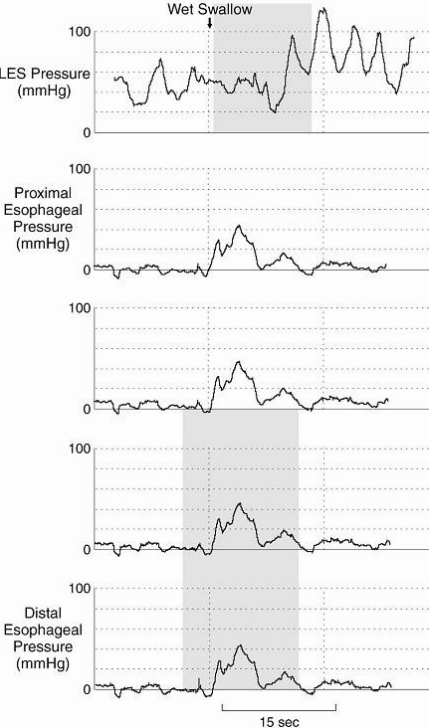
Manometry. Isobaric simultaneous esophageal body contractions with incomplete LES relations classic for the diagnosis of achalasia.

Manometry is the gold standard means for establishing the diagnosis of achalasia. Aperistalsis is always present in the esophageal body. Wet and dry swallows are followed by simultaneous contractions [1]. The amplitude of the contractions is low (10–40 mm Hg) and repetitive in most of the cases [6] (Figure 1).

The LES displays high pressure at rest and fails to relax, or relaxes only partially with swallowing (Figure 1). Up to 40% of the patients with achalasia have normal LES pressure (10–40 mm Hg); however, low pressure LES is not seen in untreated achalasia patients [47].

Vigorous achalasia was described in 1957 as a subset of achalasia with a higher contraction amplitude (>37 mm Hg), minimal esophageal dilatation, prominent tertiary contractions, and higher incidence of chest pain. A recent study comparing patients with classic and vigorous achalasia showed that the original manometric and radiographic description of vigorous achalasia is accurate; however, the incidence of the chest pain seems to be similar between the two groups [48].

### Timed barium esophagogram

**Figure 2:**
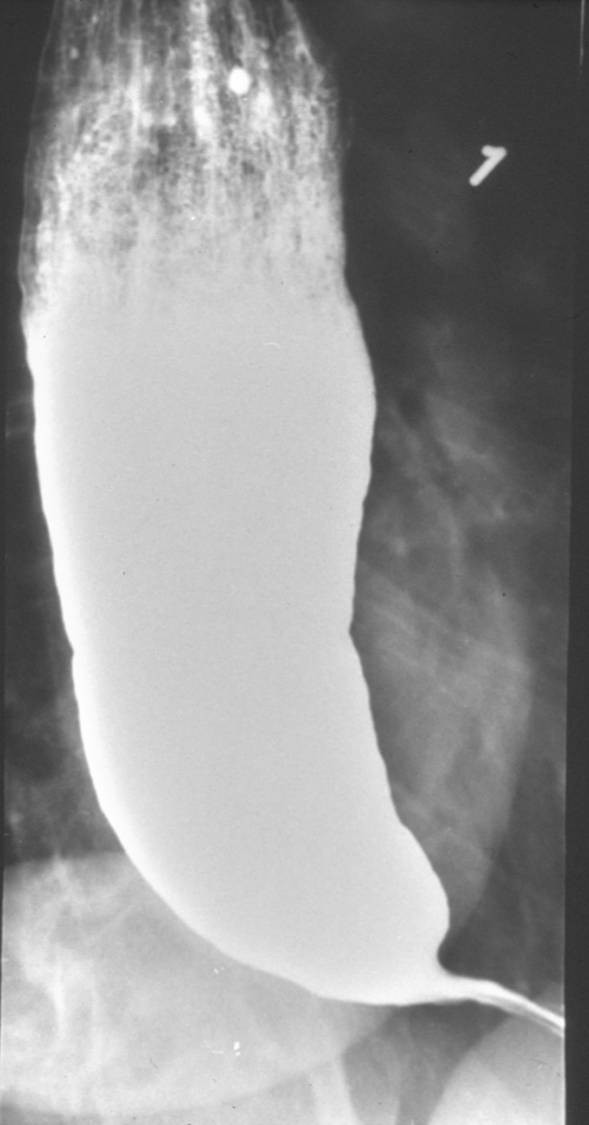
Barium swallow. Dilated esophagus with retained column of barium and "bird's beaking" suggestive of achalasia.

Barium swallow was initially used by Vantrappen *et al *[49] in achalasia patients to determine the cause of persistent symptoms after treatment with pneumatic dilation. This led to suggestion that barium esophagogram with fluoroscopy is the single best diagnostic study for achalasia. The characteristics of achalasia in barium esophagogram are the loss of primary peristalsis in the distal two third of the esophagus, poor emptying with retained food and saliva producing an air-fluid level at the top of the barium column. In the chronic stages of the disease, there is dilated esophagus or sigmoid tortuosity and, sometimes in advanced cases, massive dilatation of esophageal body [1]. The typical finding in achalasia is the presence of smooth tapering of the lower esophagus leading to a closed LES, resembling a bird's beak (Figure 2).

In 1997 de Oliveira *et al *[50] described timed barium esophagogram as a simple, noninvasive, and widely available barium technique for evaluating esophageal emptying in patients with achalasia. The films in this technique are taken at 1, 2 and 5 minutes after the last swallow of barium; the purpose of 2 min film is to assess interim emptying. The technique is simple to interpret because both radiologists and gastroenterologists can accurately assess emptying. Emptying can be assessed by the height time width of the barium column or a qualitative estimate of emptying. This method can be also used in predicting the success of treatment in patients with achalasia, which will be discussed later [12,50].

### Endoscopy

All patients with suspected achalasia should undergo upper gastrointestinal endoscopy to exclude pseudoachalasia. Pseudoachalasia results from a tumor at the esophagogastric junction, therefore, this area needs to be examined carefully during the procedure [1,3]. At endoscopy, the esophageal body may look normal, or dilated, atonic and often tortuous. The mucosa looks normal, but sometimes it is thickened or friable with even superficial ulcers secondary to chronic stasis or candida esophagitis. The LES is closed even with insufflations of air, but the endoscope can easily pass this area with gentle pressure. If a tumor is suspected because of rapid progression of symptoms, or the need of excess pressure to open the LES, repeated endoscopy examinations with biopsies and endoscopic ultrasound and CT chest are mandatory.

## Management

Despite several insights into the pathophysiologyof achalasia, the etiology of the disorder still remains unknown; thus, it is not surprising that the treatment is entirely palliative. If untreated, the disease course leads to a progressive stasis and dilation of the esophagus occurs increasing the subsequent risk of aspiration, weight loss, and malnutrition.

The objective of the current therapeutic options for achalasia is to reduce the LES tone, relieve functional obstruction to the esophageal transit, and facilitate esophageal emptying by gravity. This goal can be achieved by either non-surgical methods such as traditional muscle relaxants, chemical denervation (*i.e. *botulinum toxin), and pneumatic dilation, or surgical myotomy. These methods vary in their level of invasiveness and risk of adverse effects [10,51].

### Pharmacologic treatment

Pharmacologic agents used for achalasia are mostly smooth muscle relaxants that act by reducing LES pressure. Calcium channel blockers and long-acting nitrates are the two most common medications used [52,53]. Other less commonly used agents include anti-cholinergics (atropine, dicyclomine, cimetropium, bromide), beta-adrenergic agonists (terbutaline), and theophylline [13].

Nifedipine is one of the well-studied calcium channel blockers for the treatment of achalasia. It is available in a sublingual formulation, resulting in rapid absorption and clinical response. Studies show that the time to maximum effect for sublingual nifedipine is 20 to 45 minutes; therefore, it is recommended nifedipine (10–30 mg) to be given sublingually 30 to 45 minutes before meals and at bedtime [13]. The efficacy of nifedipine largely varies (0% to 75% in clinical trials), with side effects reported by up to 30% of the patients [10,13].

Sublingual isosorbide dinitrate is also effective in decreasing LES pressure in achalasia patients, resulting in symptom improvement in 53% to 87% of patients. The effect of nitrate is more rapid than that of nifidipine, but has a shorter duration; thus, sublingual Isordil^® ^(5 mg) is commonly administered only 10 to 15 minutes before meals [13].

In a study comparing the effect of sublingual nifedipine to sublingual isosorbide dinitrate, both drugs decreased LES pressure, but the effect of nitrate was slightly better than that of nifidipine (65% *vs. *49% respectively) [53].

Pharmacologic agents rarely yield satisfactory long-term alleviation of symptoms and are now only used in patients who are not candidates for pneumatic dilation or surgery. They also can be used during the planning stage for more effective therapy.

### Botulinum toxin treatment

Botulinum toxin (BT) was first used in achalasia patients by Pasricha and his colleagues [2,54,55]. The rational was that the injection of BT in the LES reduces the LES pressure and improves the "passive" esophageal emptying by counterbalancing the selective loss of inhibitory neurons in the myenteric plexus [2,10]. BoTx A 80–100 Units of are injected through a 5-mm sclerotherapy needle into the LES. Aliquots equaling 20 to 25 U of the toxin are injected into each quadrant of the LES.

Following single injection of BT, relief of symptoms has been reported in almost 80% of patients. After six months, 50% of the patients may remain in remission [1,2,55,56], while others will need repeated injections, or other treatment options such as pneumatic dilation (PD) or myotomy [1,55,56]. Table 1 reflects the symptom response rate as well as percent of LES pressure drop after treatment with BT over a period of 12 months in most valuable studies.

**Table 1:** Effect of botulinum toxin on achalasia

Study	Method	Number of patients enrolled	% LES pressure decreased post treatment	Remission rate at 1 months	Remission rate at 6 months	Remission rate at 12 months
Pasricha *et al*, [55]	Randomized control trial	21	33%	90%	44%	___
Fishman *et al*, [56]	Prospective study	60	___	70%	___	36%
Gordon *et al*, [75]	Prospective study	16	___	75%	48%	___
Vaezi *et al*, [2]	Randomized trial	24	1%	60%	50%	32%
Annese *et al*, [71]	Randomized trial	16	49%	100%	___	12.5%
Pasricha *et al*, [58]	Prospective study	31	45%	90%	64%	___
Martinek *et al*, [76]	Prospective cohort study	49	65%	93%	___	41%
Zaninotto *et al*, [77]	Randomized controlled trial	40	___	___	66%	34%

Only a few studies are available on the long-term efficacy of BT. Our initial randomized trial found a one year success rate of 32% in achalasia patients treated with BT [2]. Annese *et al *[57] reported a success rate of 68% at 24 months after receiving repeated BT injection, while Pasricha *et al *[58] found a 30% efficacy rate after a mean follow-up of 2 years.

Injection of BT seems to be simple and safe, without carrying any risk of perforation. Mild chest pain has been reported in a few cases shortly after the injection, but it does not usually require specific intervention. However, surgeons have also reported that myotomy is more difficult in patients with repeated BT injection due to increased adhesion of the muscular layer [51].

Post-treatment evaluations have revealed that neither pre-treatment LES pressure, amplitude of esophageal contractions, nor duration of illness could be used to predict the outcome of BT injection. Instead, young age and male gender were found to adversely affect the outcome [58,59]. Symptom relief was found to last up to 1 to 2 years with a single injection in the elderly [8,9]. Of the different BT regimen, two injections of 100 U of BT 1 month apart appears to be the most effective therapeutic schedule [57]. Seventy six percent of those responded to the first injection will respond to the second injection; however, the response to further injection usually decreases due to anti body formation against the BT protein.

Overall, BT is recommended to be most effective in elderly patients, in whom dilation or surgery represent a high risk, or in patients with co-morbid illnesses who are not candidates for PD or myotomy.

### Pneumatic dilation

Pneumatic dilation (PD) is the most effective non-surgical treatment option for patients with achalasia [6]. It uses the air to dilate the esophageal lumen and disrupt the circular muscle fibers of the LES [1]. The most commonly used balloons are nonradiopaque polyethylene Microvasive Rigiflex dilators that can be passed over a guide wire and the Witzel dilator, which is passed in a retrograde fashion over the endoscope.

The Rigiflex baloons come in three different diameters (3.0, 3.5, and 4.0 cm). No only the procedure requires sedation, but also all the PD candidates should be surgical candidates as up to 5% of patients may need surgical intervention due to perforation. The PD 3 cm is usually used first. Good to excellent relief of symptoms are reported in 50% to 93% of patients [13]. In case of symptom relapse, graded PD (increasing the size of the balloon diameter) seems to improve the clinical response.

Cumulatively, dilation with 3.0-, 3.5-, and 4.0-cm balloon diameters results in good to excellent symptomatic relief in 74%, 86%, and 90% of treated patients, respectively with an average follow-up of 1.6 years [13]. Of the few prospective studies on the long-term efficacy of PD, Eckardt *et al *followed 54 consecutive patients treated with PD every 2 years [59] and reported when follow-up on the patients was extended further (for a median of 13.8 years), an overall 5 year remission of 40% and a 10-year remission rate of 36% was observed. They found that repeated dilations only mildly improved the clinical response [60]. Other studies have shown good to excellent symptom improvement in 50–89% of patients over a mean follow-up of 4 years [13,61,62].

Although PD is an outpatient procedure that is performed under standard endoscopic sedation, there is still a small but significant risk of perforation. The cumulative perforation rate with Rigiflex balloons is reported to be 2% [13].

Current knowledge has let us to make some predictions on the outcome of pneumatic dilation. The failure rate of PD is suggested to be higher in children and adolescents than in elderly patients. Our recent study also showed a predictor role for age and gender, as young male patients do not do as well with the 3.0 cm Rigiflex balloon dilation [63]. Vela *et al *[64], later confirmed these observations. There is also evidence that a post-treatment LES pressure of less than or equal to 15 mmHg correlates with successful treatment outcome [61]. The role of radionuclide studies in predicting the outcome of PD is in doubt. Esophageal emptying in radionuclide studies of two patients post-therapy showed a correlation with symptom improvement [65]; however, larger studies failed to establish a definite role for this method in predicting the success of the treatment [59,66,67].

Our recent studies [12,61,68,69] suggest timed barium esophagogram as a better predictor of treatment success after PD. We have found that in almost 70% of the patients, the height of the barium column at 5 minute post-therapy correlates with symptom improvement (concordant group), while in others esophageal emptying was poor despite reports of excellent symptom relief (discordant group). Nearly all patients in discordant group failed the treatment within 1 year after treatment, while 77% of the concordant group were still in symptom remission after 6 years of follow-up [12]. Therefore, it is suggested that the timed barium esophagogram not only assesses treatment shortly after therapy, it can also predict the poor response to the treatment if the patient has retained barium post pneumatic dilation.

### Surgical cardiomyotomy

Surgical management of achalasia involves performing a Heller myotomy (HM), combined with an antireflux procedure (Toupet or Dor). For many years this operation has been performed through a laparotomy, often with the addition of a fundoplication, or through a left thoracotomy [70]. However, the advent of minimally invasive surgery (laparoscopic myotomy) has resulted in several advantages such as superior visualization of the gastroesophageal junction, the ability of adding an antireflux procedure, a shorter hospital stay (2 days compared with 5–7 days), reduced morbidity, and quicker return to daily activity [13,70].

A cumulative good to excellent clinical response rate of 94% has been reported for laparoscopic cardiomyotomy over a short period of time. Studies on long-term outcome of myotomy are summarized in Table 2. The major disadvantages of myotomy are incomplete myotomy and the possibility of significant GERD. Table 2 also shows the rate of developing GERD after myotomy in the most valuable studies reported.

**Table 2:** Long-term result of laparoscopic myotomy with fundoplications

Study	Method	Method of surgery	Number of patients enrolled	Length of follow-up	Good to excellent response	GERD* complication
Bessell *et al*, [78]	Prospective	Laparoscopic HM**	167	5 years	77%	Not mentioned
Vella *et al*, [64]	Retrospectivecohort	88% Laparoscopic and 12% open HM	73	6 years	57%	36%
Dang *et al*, [79]	Retrospective	81% Laparoscopic and 9% open HM	22	3 years	76%	Not mentioned
Raiser *et al*, [80]	Retrospective	Laparoscopic or thoracoscopic HM	35	1–4 years	97%	Not mentioned
Hunt *et al*, [81]	Retrospective	Laparoscopic HM	70	2.9 years	81%	4.5%
Frantzides *et al*, [82]	Retrospective	Laparoscopic HM	53	3 years	92%	9%
Zaninotto *et al*, [83]	Prospective	Laparoscopic HM	100	2 y	92%	7%

*GERD Gastroesophageal reflux disease; **HM Heller myotomy

The preferred treatment after a failed myotomy is PD. A recent study on untreated achalasia patients and patients with failed myotomy reports no increased risk of perforation with performing PD after Heller myotomy. However, this study also indicates that despite lower LES pressure, patients undergoing PD after failed myotomy do not do as well as untreated cases [11].

Finally, laparoscopic surgery on achalasia patients that used to be only done on those that relapsed after graded pneumatic dilation is increasingly being performed on healthy achalasia patients younger than 40 years. In addition, surgery may be indicated as the first line therapy for patients with tortuous esophagus, esophageal diverticula, or previous surgery on the gastroesophageal junction.

### Comparison of the procedures

Several randomized trials suggest that pneumatic dilation is more effective than botulinum toxin [2,71,72]. Our study, which was one of the largest studies comparing the outcome of two therapies, suggested that both therapies are effective at 1 month, but PD results in significantly better symptom improvement at 12 months compared with BT (70% *vs. *32% respectively) [2]. These findings indicate that botulinum toxin is inferior to pneumatic dilation for sustain symptom relief. A study on cost-effectiveness of treatments also suggest that in long-term PD is a less cost-effective treatment for achalasia compared to BT [73].

Comparison of PD and HM also shows that there is no difference in the early outcome of these treatments, and the success rate of both methods decreases over time (90% *vs. *89% respectively at 6 months, to 44% *vs. *56% at 6 years) [64]. Studies also suggest that laparoscopic myotomy is not a cost-effective therapy as the initial cost is too high [73]. However, it is an effective treatment modality in patients with achalasia who have failed to respond to PD, as the 10-year remission rate in these patients following myotomy is shown to be 77% comparing to 72% and 45% in patients "successfully" treated with a single PD and patients undergoing several dilations respectively [74].

Therefore, to make a decision on the treatment of achalasia, patients should be involved in an honest discussion on the efficacy, possible complications, as well as cost-effectiveness of each of the therapeutic options, and patients' informed decision should guide therapy.

## Conclusion

Achalasia is a motor disorder of the esophagus characterized by dysphagia, regurgitation, and chest pain. Although it cannot be permanently cured, excellent palliation is available in over 90% of patients. As a result of the advances in pneumatic dilation and laparoscopic Heller myotomy, most patients with achalasia can now choose between these two treatments. The injection of botulinum toxin endoscopically into the LES is usually reserved for elderly, or patients who are not candidates for pneumatic dilation or surgery. In patients unresponsive to graded pneumatic dilation, esophageal myotomy via the laparoscopic method should be performed. In myotomy failures, repeat pneumatic dilation should be attempted.
